# Anogenital distance as a biomarker for endometriosis in adolescents: a prospective, matched case–control study

**DOI:** 10.3389/frph.2026.1951393

**Published:** 2026-09-17

**Authors:** Jessica Y. Shim, Ava Scatoni, Daniel W. Cramer, Steven J. Staffa, Marc R. Laufer, Elizabeth R. Boskey

**Affiliations:** 1 Division of Gynecology, Department of Surgery, Boston Children’s Hospital, Boston, MA, United States; 2 Department of Obstetrics, Gynecology and Reproductive Biology, Harvard Medical School, Boston, MA, United States; 3 Department of Obstetrics and Gynecology, Brigham and Women’s Hospital, Boston, MA, United States; 4 Department of Anesthesiology, Boston Children’s Hospital, Boston, MA, United States

**Keywords:** adolescence, anogenital distance, biomarker, case–control study, endometriosis

## Abstract

**Objectives:**

To determine whether anogenital distance (AGD), a potential non-invasive biomarker reflecting prenatal androgen exposure, differs between adolescents with laparoscopically confirmed endometriosis and matched controls.

**Methods:**

This IRB-approved, matched case–control study was conducted at a single pediatric hospital between March 2024 and June 2025. Twenty adolescents aged 13–25 years were enrolled: 10 with laparoscopically confirmed endometriosis and 10 controls (three asymptomatic, seven laparoscopically confirmed without endometriosis), matched by age (±1 year) and body mass index (BMI) (±2 kg/m^2^). AGD was measured under anesthesia from the clitoral surface to the anus (AGD-AC) and from the posterior fourchette to the anus (AGD-AF). Within-pair mean differences were calculated using paired *t*-tests.

**Results:**

The mean AGD-AC was 9.4 ± 1.2 cm in cases versus 9.7 ± 1.4 cm in matched controls; the mean AGD-AF was 3.5 ± 0.5 cm in cases versus 3.2 ± 0.8 cm in matched controls. Paired *t*-tests showed no significant differences in either [mean difference AGD-AC = 0.297, 95% CI (−0.85 to 1.44) *p* = 0.57; AGD-AF = −0.327, 95% CI ( −0.88 to 0.23) *p* = 0.22]. Substantial overlap was observed across groups, and no significant differences were identified for either AGD measure.

**Conclusion:**

We did not detect a difference in AGD measurements between adolescents with laparoscopically confirmed endometriosis and age and BMI-matched controls; however, with only 10 matched pairs, this study was powered to detect only a large effect, and therefore, these null findings should be interpreted cautiously. Larger longitudinal studies are warranted to assess whether AGD correlates with disease onset, severity, or progression.

## Introduction

Endometriosis is a chronic gynecologic disorder characterized by the presence of endometrial-like tissue outside the uterine cavity. Evidence increasingly supports that endometriosis can begin in adolescence, presenting with symptoms such as dysmenorrhea and pelvic pain. Similar to adults, adolescents often experience diagnostic delays, largely because of reliance on laparoscopy for confirmation (1). Early diagnosis is crucial for initiating timely management, preventing disease progression, and mitigating long-term sequelae, highlighting the need for non-invasive diagnostic markers.

One proposed biomarker for endometriosis is anogenital distance (AGD)—the distance from the anus to the clitoral surface (AGD-AC) or the posterior fourchette (AGD-AF). AGD is a sexually dimorphic anatomical trait determined *in utero* and influenced by prenatal androgen exposure; males, with a higher testosterone-to-estrogen ratio, generally develop a longer AGD, whereas females, with a lower ratio, have a shorter AGD (2). Recent research has proposed AGD as a marker of fetal endocrine disruption and associated reproductive disorders, including endometriosis (3). Several studies report that women with endometriosis, particularly those with deep infiltrating disease, have a shorter AGD, suggesting estrogen excess relative to testosterone during fetal development (4–6). However, these studies focus on adults, often with advanced-stage disease, leaving adolescent populations unexamined (5).

Adolescents represent a critical population for studying AGD, as adolescence represents an early stage in the disease trajectory or adolescents may exhibit different phenotypic characteristics. Diagnostic delay is common among adolescent patients and may worsen disease progression, contributing to chronicity of pain symptoms and significant physical and psychological impact (7). Thus, early intervention and treatment for adolescents may alter long-term health and psychosocial outcomes. If AGD is associated with endometriosis in this age group, it could serve as a low-cost, non-invasive biomarker for early risk stratification and management. We therefore aimed to evaluate whether AGD differs between adolescents with endometriosis and matched controls.

## Materials and methods

We conducted a prospective, matched case–control study through the pediatric and adolescent gynecology division at a tertiary pediatric hospital between March 2024 and June 2025. The institutional review board approved the study protocol, and informed consent (and assent when appropriate) was obtained from all participants. A small monetary token was provided for participation.

Eligible participants were adolescents aged 13–25 years undergoing laparoscopy or another procedure under general anesthesia. Cases were adolescents with laparoscopically confirmed endometriosis. Controls were asymptomatic adolescents undergoing unrelated procedures (e.g., hymenal abnormality correction, ovarian cyst removal) with no history of dysmenorrhea, chronic pelvic pain, or a suspected or confirmed diagnosis of endometriosis, or who underwent laparoscopy and did not have confirmed endometriosis. Because adolescents who were laparoscopically confirmed to not have endometriosis were infrequently available within the recruitment window, controls also included asymptomatic adolescents undergoing anesthesia for non-gynecologic indications, allowing an adequately sized comparison group to be recruited.

Controls were matched 1:1 by age (within ±1 year) and body mass index (BMI; within ±2 kg/m^2^) to minimize confounding related to growth and body composition. Adolescents with ambiguous genitalia, intersex conditions, prior hormonal treatment affecting genital development, or polycystic ovary syndrome were excluded. Intraoperative data collected included the presence, location, and staging of endometriosis according to the revised American Society for Reproductive Medicine (rASRM) classification system (8).

AGD was measured in dorsal lithotomy position using a measurement tape by trained providers. Two AGD measurements were taken for each participant **(**Figure 1**)**:

- AGD-AC: Distance from the clitoral surface to the anterior verge of the anus
- AGD-AF: Distance from the posterior fourchette to the anterior verge of the anus

**Figure 1:**
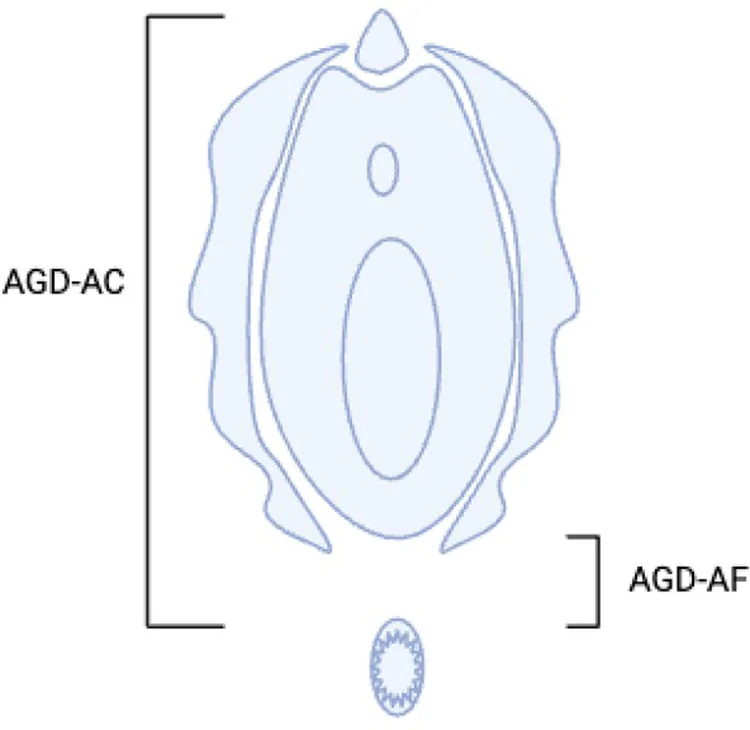
An illustration of landmarks for anogenital distance measurements. AGD-AC, from the anterior clitoral surface to the upper verge of the anus; AGD-AF, from the posterior fourchette to the upper verge of the anus.

Measurements were obtained under general anesthesia and prior to surgery, ensuring that the entire surgical team remained blinded to disease status at the time of measurement. Each measurement was taken three times consecutively. The average of the three was used as the primary value for analysis, and the within-subject standard deviation was calculated to assess measurement reliability.

### Sample size and power calculation

A power analysis was conducted using G*Power version 3.1 (University of Düsseldorf, Germany). Assuming previously reported AGD-AC means of 10.09 cm (SD = 2.06) versus 8.38 cm (SD = 1.29) (5), a sample size of 17 participants per group (*N* = 34 total) would provide 80% power to detect a statistically significant difference using a two-sided *t*-test with alpha = 0.05. This corresponds to a standardized effect size of 1.0. For matched groups with paired analysis (paired *t*-test), the required sample size decreased to 10 participants per group (*N* = 20 total) to achieve 80% power, assuming a moderate within-pair correlation of 0.5. Thirty-four participants were recruited in total, of whom 20 were successfully matched (22 cases and 12 controls). The 20 matched participants (10 cases and 10 controls) were successfully matched 1:1 on age and BMI and constituted the primary matched analysis, whereas the remaining 14 participants (12 cases and two controls) could not be matched and, together with the matched participants, were included in the secondary full-population analysis (*N* = 34).

### Statistical analysis

Continuous variables for the matched population (*n* = 20) were reported as means with standard deviations (SD) and compared using paired or independent *t*-tests as appropriate. Categorical variables were compared using Fisher's exact test. Within-subject variability was compared using within-subject coefficient of variation across repeated measures (9). Robustness analyses to look at measures in the full population (*n* = 34) were run as analyses of variance to compare AGD-AC and -AF across case and control status, with an additional Pearson's correlation testing to examine whether there was a relationship between AGD and BMI in the full population. We ran multiple regressions on AGD-AC and -AF with case status, age, and BMI as an additional sensitivity test on the full population to determine whether age was either associated with AGD or affected the association between case status and AGD. A two-tailed *p*-value < 0.05 was considered statistically significant. Analyses were performed using Stata 18–19.x (StataCorp LLC; College Station, TX, USA).

## Results

Ten adolescents with endometriosis were matched to 10 asymptomatic controls. The mean age was 15.3 ± 1.6 years among cases and 14.8 ± 1.4 years among controls. The mean BMI was 23 ± 5.9 in cases and 23 ± 5.4 in controls. All participants were nulliparous and postmenarchal (Table 1).

**Table 1:** Clinical characteristics and anogenital distance (AGD) measurements from the clitoral surface to the anus (AGD-AC) and from the posterior fourchette to the anus (AGD-AF)..

Demographics and AGD measurements	Controls (*n* = 10)	Cases (*n* = 10)	*p*-Value
Age at surgery (years)	14.8 (1.4)	15.3 (1.6)	0.40
BMI at surgery (kg/m^2^)	23 (5.4)	23 (5.9)	0.89
AGD-AC mean (cm)	9.7 (1.4)	9.4 (1.2)	0.57
AGD-AF mean (cm)	3.2 (0.8)	3.5 (0.5)	0.22
Surgery indication, *n* (%)			
Pelvic pain/dysmenorrhea	1 (10)	10 (100)	
Hymenectomy/hymen abnormality	3 (30)	0 (0)	
Ovarian cyst	6 (60)	0 (0)	
Endometriosis stage, *n* (%)			
1	–	9 (90)	
2	–	1 (10)	
Endometriosis locations, *n* (%)			
Posterior cul-de-sac	–	10 (100)	
Left pelvic side wall	–	8 (80)	
Right pelvic side wall	–	6 (60)	
Anterior cul-de-sac	–	7 (70)	
Endometrioma	–	0 (0)	
Adenomyosis	–	0 (0)	
Other^a^	–	2 (20)	
Deep endometriosis	–	0 (0)	

Cohort matched on BMI and age.

a Left uterosacral ligament; left ovary to utero-ovarian ligament, bladder peritoneum.

Among matched controls, indications for surgery included hymenal abnormalities (30%), benign ovarian cysts (60%), and pelvic pain (10%). Patients with the ovarian cyst and pelvic pain were confirmed not to have endometriosis during their laparoscopy. All patients had chronic pelvic pain or dysmenorrhea, and all were diagnosed with endometriosis laparoscopically. Nine (90%) had stage I and one (10%) had stage II disease. All had superficial peritoneal endometriosis, and no participants had endometriomas or deep infiltrating disease.

The mean AGD-AC was 9.4 ± 1.2 cm in adolescents with endometriosis and 9.7 ± 1.4 cm in controls. Paired *t*-tests showed no significant difference {mean difference [0.297, 95% CI ( −0.85 to 1.44) *p* = 0.57]}. The mean AGD-AF was 3.5 ± 0.5 cm in cases compared with 3.2 ± 0.8 cm in controls, and differences on paired *t*-tests were not significant {mean difference [−0.327, 95% CI ( −0.88 to 0.23) *p* = 0.22]}. The within-subject coefficient of variation was 0.015 [95% CI (0.012–0.017)] for AC and 0.045 [95% CI (0.037–0.054)] for AF.

To determine whether a failure to account for body size might be one reason that an association was seen in other study populations, we also ran the same series of tests on the full population who were recruited for the study (Table 2). In the full population, the controls were slightly but significantly younger [mean age 14.8 (1.6) vs. 16.8 (2.2), *p* = 0.02], but there were no differences in BMI or its constituent components of height or weight. In this group, the mean AGD-AC was 10.0 ± 1.7 cm in adolescents with endometriosis and 9.8 ± 1.3 cm in controls (*p* = 0.82). The mean AGD-AF was 3.5 ± 0.8 cm in cases compared with 3.3 ± 0.8 cm in controls (*p* = 0.59)—reducing the difference between average measurements. In the full population of adolescents, we also observed that the mean AGD-AC (Pearson's correlation coefficient=0.78, *p* < 0.001) and AGD-AF (coeff.=0.65, *p* < 0.001) were highly correlated with BMI. When adjusted regressions were run including case status, BMI, and age, we found that only BMI was associated with either AGD-AC or AGD-AF (data not available) and age did not affect the association between either BMI or case status and AGD.

**Table 2:** Sensitivity analysis looking at clinical characteristics and anogenital distance (AGD) measurements from the clitoral surface to the anus (AGD-AC) and from the posterior fourchette to the anus (AGD-AF) for an entire recruited sample.

	Controls (*n* = 12)	Cases (*n* = 22)	*p*-Value
Age at surgery (years)	14.8 (1.4)	16.8 (2.2)	**0**.**02**
BMI at surgery (kg/m^2^)	24.6 (7.3)	24.7 (7.9)	0.97
AGD-AC mean (cm)	9.8 (1.3)	10.0 (1.7)	0.82
AGD-AF mean (cm)	3.3 (0.8)	3.5 (0.8)	0.59
Surgery indication			
Pelvic pain/dysmenorrhea	1 (8)	20 (91)	
Hymenectomy/hymen abnormality	4 (33)	1 (4)	
Ovarian cyst	7 (58)	3 (14)	
Endometriosis stage			
1	–	18 (82)	
2	–	2 (9)	
3	–	1 (4)	
4	–	1 (4)	

The bold value is statistically significant.

## Discussion

In this matched case–control study of adolescents, AGD did not differ between those with laparoscopically confirmed endometriosis and asymptomatic matched controls. Both AGD-AC and AGD-AF measurements were comparable across groups, and within-subject variable was also minimal, suggesting measurement reliability. These findings suggest that AGD may not serve as a clinically useful, non-invasive biomarker in this population.

Our findings contrast with those of some prior adult studies that reported a shorter AGD with endometriosis. For example, Mendiola et al. (4) observed an approximately 17 mm shorter AGD-AC difference in affected women, whereas Sánchez-Ferrer et al. (6) found even greater differences among those with deep infiltrating endometriosis. Likewise, Crestani et al. (5) confirmed a shorter AGD in women with surgically confirmed endometriosis. However, these findings were not universal. Khan et al. (10) found no significant differences in AGD between controls and women across all rASRM stages, and Harth et al. (11) reported no correlation between magnetic resonance imaging-based AGD and surgical diagnosis.

Several factors may account for the discrepancy between our findings and those of the prior adult studies, which found an association between AGD and endometriosis. First, our cohort represented almost entirely stage I disease, and it is possible that AGD differences may emerge only with advanced phenotypes. Adolescents may present earlier in the disease course and exhibit a different distribution of endometriosis phenotypes. A study examining age-related phenotype variation found that in women aged 24 years or younger, the frequency of deep infiltrating disease was lower, while isolated superficial lesions were more prevalent (12).

Second, AGD, although determined *in utero*, may be influenced by pubertal development or external factors, potentially obscuring differences in younger populations whose bodies may still be growing and changing. This hypothesis is not clearly supported or refuted by our data; in our unmatched populations, despite no significant differences in height or weight—only age—the direction of the AGD-AC difference switched from that which was seen in the matched population. Because neither estimate was statistically significant and confidence intervals were wide, we interpret this as reflecting the “noise” that would be expected when there is no true association. In addition, although general anesthesia provided a controlled setting for the AGD measurements, differences in muscular relaxation or tissue tone may have still contributed to variability.

Finally, AGD may not be an accurate causal biomarker but rather a surrogate for exposure to hormonal or environmental factors that are associated with, but not determinative of, endometriosis development—or even of differences in body habitus (e.g., BMI) that were not always accounted for in a previous study. Given that, in our analysis, AGD was highly correlated with BMI and BMI is potentially correlated with endometriosis (13), it is possible that body habitus may confound previous results wherein the researchers did not consider height and weight as part of their analyses.

Importantly, a lack of association between AGD and endometriosis does not necessarily refute the underlying hypothesis that prenatal hormonal influences affect endometriosis risk. However, it calls into question the utility of AGD as a useful marker for those changes in the context of diagnosing endometriosis—particularly without some way to address the confounding relationship that BMI potentially has with both AGD and endometriosis.

This study has several strengths. To our knowledge, it is the first to evaluate AGD in an adolescent population with confirmed endometriosis, with rigorous matching, blinded standardized measurements, and reporting of within-subject variability. The initial study design sought participants aged 13–25 years to capture both adolescents and young adults; ultimately, all participants in the matched sample were under 18 years of age (whereas four in the full sample were 20 years of age and above), providing, to our knowledge, the first adolescent sample in literature. By recruiting participants from a pediatric tertiary care center, we captured a clinically relevant population that is highly likely to benefit from a non-invasive screening tool. We also matched by age and BMI in order to address concerns that body size might play a role in previously observed associations that did not consistently adjust for body size. The primary limitation of this study is its small sample size and the resulting imprecision in effect estimates. Although our power analysis suggested 10 matched pairs would be sufficient to detect a large effect size, this study was not powered to detect smaller or more subtle differences. With *N* = 10 matched pairs, alpha = 0.05, and an assumed within-pair correlation of 0.5, this study had only 80% power to detect a standardized (paired) effect size of approximately 1.0—equivalent to an AGD-AC difference roughly of 1.3 cm given the SDs observed in our sample. The actual effect size in a predominantly early-stage adolescent population may be substantially smaller than thresholds identified in studies on adults, meaning a true but modest association could have gone undetected. The null findings should therefore be interpreted cautiously. In addition, our sample size correlations were, by necessity, based on adult samples even though this study was performed on adolescents. We also did not stratify by pubertal stage (although all patients were postmenarchal) or hormone exposure history (e.g., use of combined oral contraceptives), which have not been explored but may influence measurements. Over half of controls underwent surgery for benign ovarian cysts; because ovarian cysts may reflect an altered hormonal milieu, this control composition may not represent a neutral comparison group, and we cannot exclude the possibility that this introduced additional unaccounted for variability in AGD. The cross-sectional nature of the study also prevents us from assessing the predictive value of AGD longitudinally—whether AGD might indicate future risk before symptoms or clinical disease develops. Our cohort was almost entirely stage I, superficial peritoneal disease, with no deep infiltrating endometriosis or endometriomas represented; because prior studies on adults reporting AGD differences were often enriched with more advanced disease, our findings should not be extrapolated to adolescents with deep infiltrating disease or endometriomas, and the apparent discrepancy with some of the adult literature may partly reflect this difference in disease severity rather than a true absence of association.

In conclusion, in this small, matched cohort of 10 case–control pairs, we did not detect a significant difference in AGD between adolescents with endometriosis and age- and BMI-matched controls. Because this study was powered to detect only a large effect and was underpowered to exclude smaller, potentially clinically relevant differences, these null findings should be interpreted cautiously. Nonetheless, further research is warranted to explore whether AGD could contribute to a broader composite risk model alongside other biomarkers, clinical symptoms, or imaging findings. Future studies should consider larger, multicenter adolescent cohorts with a broader spectrum of disease severity but continue to pay careful attention to BMI given the strong correlation that we observed between the two measurements. Longitudinal designs following individuals from early adolescence through adulthood could help determine whether AGD predicts later development of endometriosis or correlates with disease progression.

## Data Availability

The raw data supporting the conclusions of this article will be made available by the authors, without undue reservation.
